# Supplementary material for: Perivascular niche cells sense thrombocytopenia and activate hematopoietic stem cells in an IL-1 dependent manner
Source: Nat Commun. 2023 Sep 28;14:6062. doi: 10.1038/s41467-023-41691-y (PMC10539537; doi:10.1038/s41467-023-41691-y)
Supplement: Supplementary file 1 — Supplementary Information File [file 41467_2023_41691_MOESM1_ESM.pdf]

Supplementary Figure 1

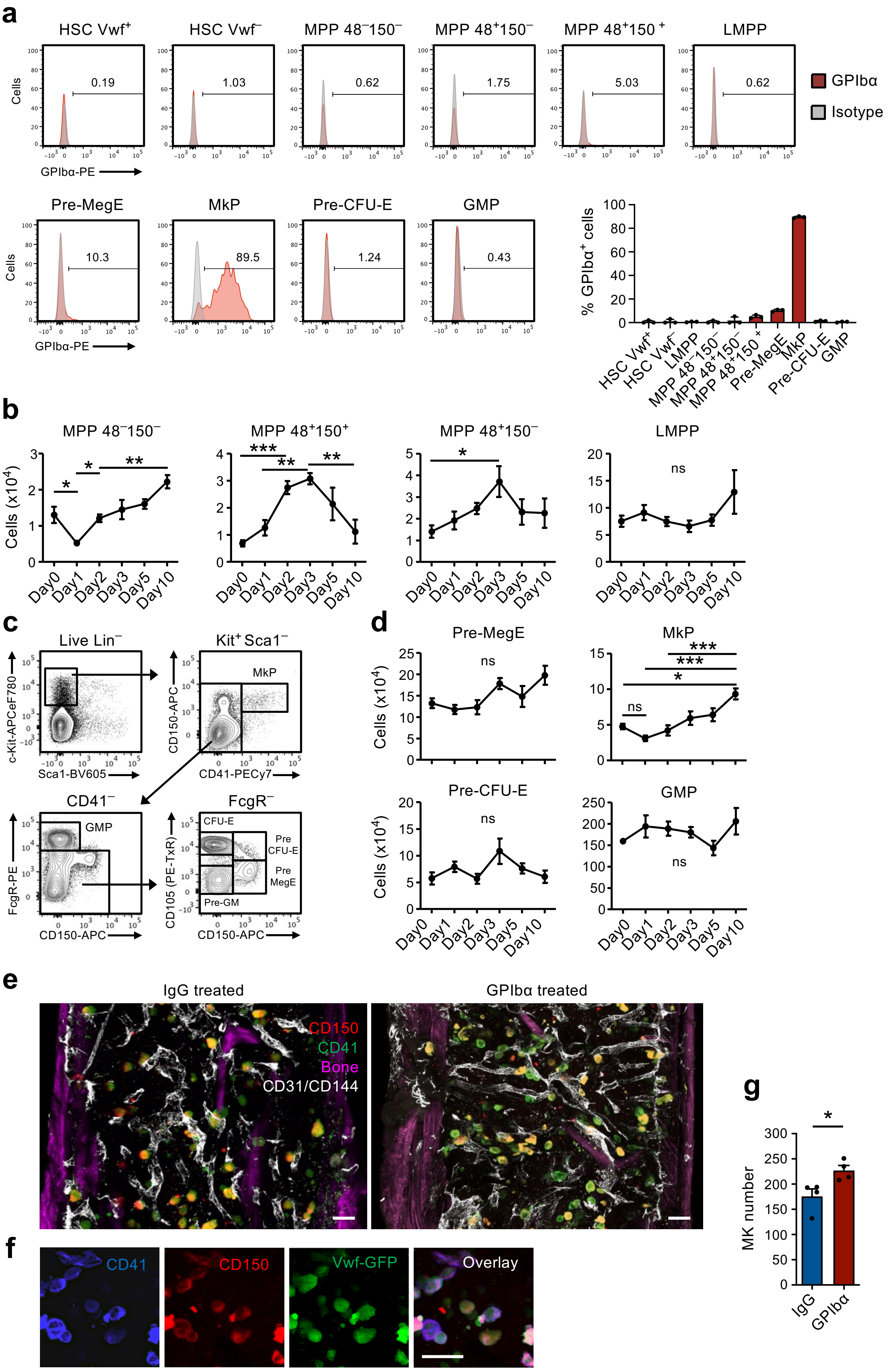

Supplementary Figure 1. Kinetics of hematopoietic stem and progenitor cells after acute platelet depletion. (Related to main Figure 1)

(a) FACS analysis of GPIbα/CD42b expression on Vwf^{+} and Vwf^{-} HSCs, multipotent progenitors and myeloid progenitors. Data shows representative histograms (numbers indicate the average frequency of mice analyzed) and mean ±SD values of 3 mice analyzed in 1 experiment representative of 3 independent experiments.

(b-d) Numbers of multipotent progenitors **(b)** and myeloid progenitors **(c-d)** at the indicated time points post platelet depletion with GPIbα antibody. **(c)** Representative FACS profiles and gating strategy of myeloid progenitors. Data represent mean ±SEM of 8 (Day0), 8 (Day1), 7 (Day2), 5 (Day3), 6 (Day5) and 5 (Day10) mice from 9 independent experiments **(b)**, and 3 (Day0), 5 (Day1), 7 (Day2), 5 (Day3), 6 (Day5) and 5 (Day10) from 6 independent experiments **(c-d)**.

(e-g) Quantification of Mk numbers in bone marrow by whole mount imaging of sternum. **(e)** Detail of sternum segments isolated from mice 1 day post IgG or GPIbα antibody administration. $CD41^{+}CD150^{+}$ Mk localization in relation to $CD31^{+}/CD144^{+}$ endothelial cells and bone detected by second-harmonic generation signal. For quantification Mks were defined as $CD41^{+}CD150^{+}Vwf\text{-}GFP^{+}$ cells >20µm in diameter **(f)**. **(e-f)** Scale bars represent 70µm. **(g)** Number of Mks per sternum segment. Data represent mean ±SD of 2 sternum segments isolated from each of 2 mice per condition and analyzed in 2 independent experiments. ***, p<0.001; **, p<0.01; *p<0.05; ns, non-significant (p>0.05), (**b-d,** 1-way ANOVA with Tukey's multiple comparisons; **g**, two-sided t-test).

Supplementary Figure 2

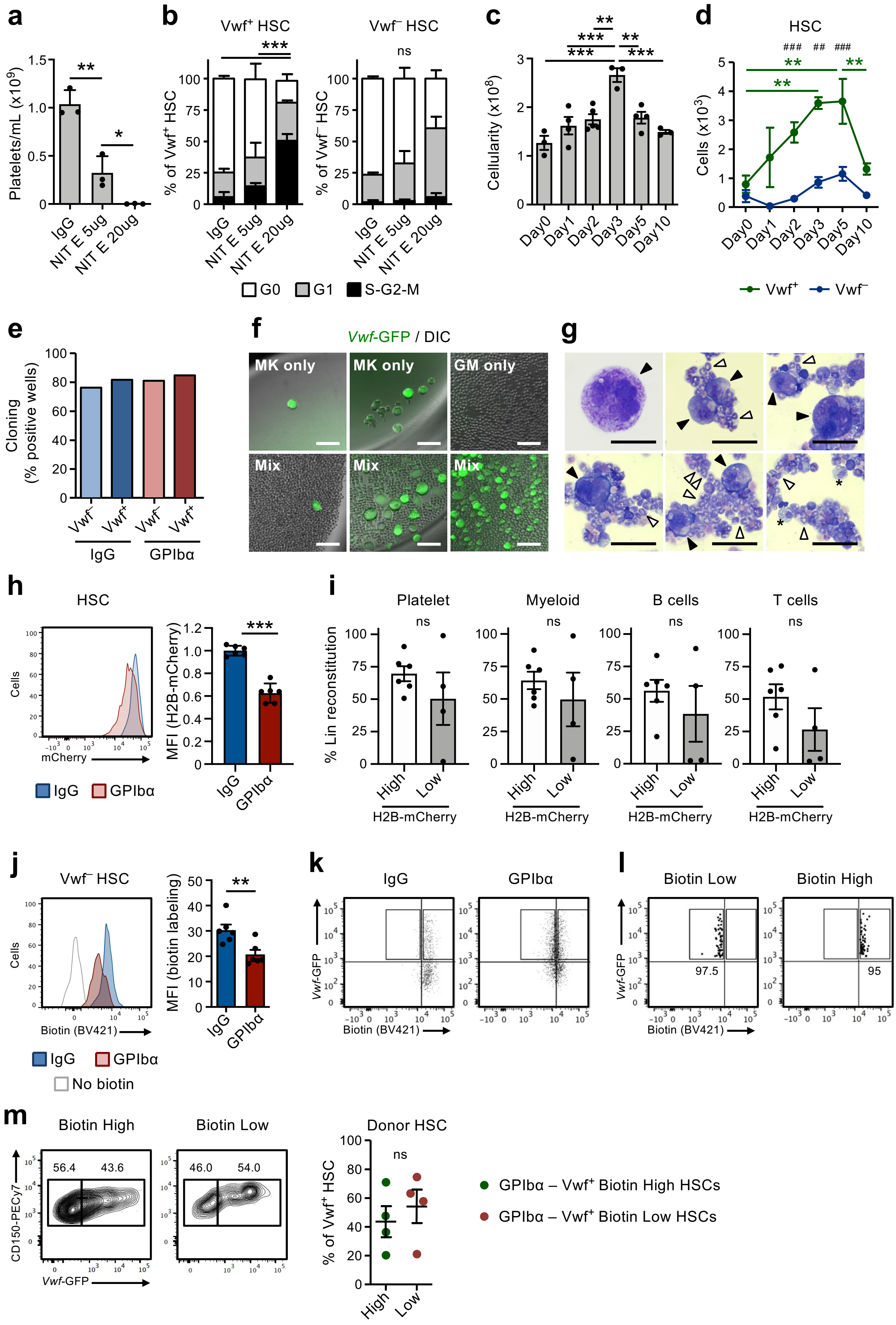

Supplementary Figure 2. Platelet depletion mobilizes Vwf⁺ HSCs and increases their megakaryocyte differentiation efficiency. (Related to main Figure 1)

(a-b) Platelet depletion efficiency **(a)** and cell cycle phase distribution of Vwf⁺ (left) and Vwf⁻ (right) HSCs **(b)** 1 day post intravenous administration of an alternative monoclonal antibody against GPIbα (NitE). Data represent mean ±SD of 3 mice per group in 2 independent experiments. Statistics in **(b)** refer to S-G2-M cell cycle stage. ****p*<0.001; ***p*<0.01; **p*<0.05; ns, non-significant (p>0.05) assessed using 1-way ANOVA **(a)** or 2-way ANOVA **(b)** with Tukey's multiple comparisons.

(c-d) Transient extramedullary hematopoiesis in spleen post platelet depletion (GPIbα). Kinetics of spleen cellularity (**c**) and absolute numbers of Vwf⁺ and Vwf⁻ HSCs in spleen (**d**) at the indicated time points post platelet depletion. In **(c)** data represent mean ±SEM of 3 (Day0), 4 (Day1), 5 (Day2), 3 (Day3), 4 (Day5) and 3 (Day10) mice in 4 independent experiments; ****p*<0.001; ***p*<0.01; **p*<0.05 (1-way ANOVA with Tukey's multiple comparisons). In **(d)** data represent mean ±SEM of 2 (Day0), 3 (Day1), 5 (Day2), 3 (Day3), 4 (Day5) and 3 (Day10) mice in 4 independent experiments; **, *p*<0.01 for Vwf⁺ HSCs; numbers of Vwf⁻ HSCs do not significantly change post platelet depletion and Vwf⁻ HSC (1-way ANOVA with Tukey's multiple comparisons); ##, *p*<0.01 and ###, *p*<0.001 for the comparison of Vwf⁺ vs. Vwf⁻ HSCs (2-way ANOVA with Sidak's multiple comparisons).

(e-g) Mk-GM lineage potential analysis of single Vwf⁻ and Vwf⁺ HSCs isolated from mice 16hrs post administration of IgG or GPIbα antibodies. **(e)** Cloning frequency. Data from 138, 364, 147 and 451 single cell-derived colonies analyzed, respectively, from 5 biological replicates in 4 independent experiments. **(f)** Wells containing Mks and or myeloid/GM cells were scored on an inverted microscope. Mk cells were defined as big *Vwf*-GFP⁺ cells. Images depict examples of Mk only, GM only and Mk/GM colonies. Scale bars represent 100µm. **(g)** May-Grunwald-Giemsa staining of cytospin slides showing cells with typical Mk (black arrow heads), neutrophil (white arrow heads) or monocytic (asterisk) morphology from day 8 cultures. Scale bars represent 50µm.

(h) H2B-mCherry dilution analysis of HSCs 3 days post IgG or GPIbα treatment. Representative plot (left) and mean ±SD MFI (normalized for MFI of IgG control; right) from 6 mice per group in 3 independent experiments. ***, *p*<0.001 (two-sided t-test).

(i) Long-term reconstitution (16 weeks) of platelet, myeloid and lymphoid cell lineages in blood by H2B-mCherry High and Low HSC fractions 3 days post platelet depletion. Data represent mean ±SEM of 4 and 6 donors of H2B-mCherry Low and High HSCs, respectively, each transplanted into 2 recipients, in 3 independent experiments. Statistical significance was assessed using two-sided t-test.

(j) Biotin proliferation analysis of *Vwf*-GFP⁻ HSCs 2 days post IgG or GPIbα treatment. Representative plot (left) and mean ±SD MFIs (normalized for MFI of No biotin labelling control; right) from 6 mice per group in 3 independent experiments. Statistical significance assessed using two-sided t-test.

(k-l) Representative FACS gating strategy **(i)** and sorting purity analysis **(j)** for FACS sorting of Biotin high and Biotin low Vwf⁺ HSCs 2 days post GPIbα treatment. **(j)** Numbers in plots indicate the average purity from 4 purity tests in 2 independent experiments.

(m) Frequency of Vwf⁺ HSCs within the donor HSC compartment (Left, representative FACS profiles; Right, combined data) 16 weeks post transplantation of biotin high and biotin low *Vwf*-GFP⁺ HSC fractions, isolated from mice 2 days post platelet depletion. Data represent mean ±SEM of 4 donors in 2 independent experiments. Each dot in the graphs represents the mean of 2 recipient mice transplanted per donor. Numbers in FACS plots are averages of all mice analyzed. ns, non-significant (p>0.05), assessed using two-sided t-test.

Supplementary Figure 3

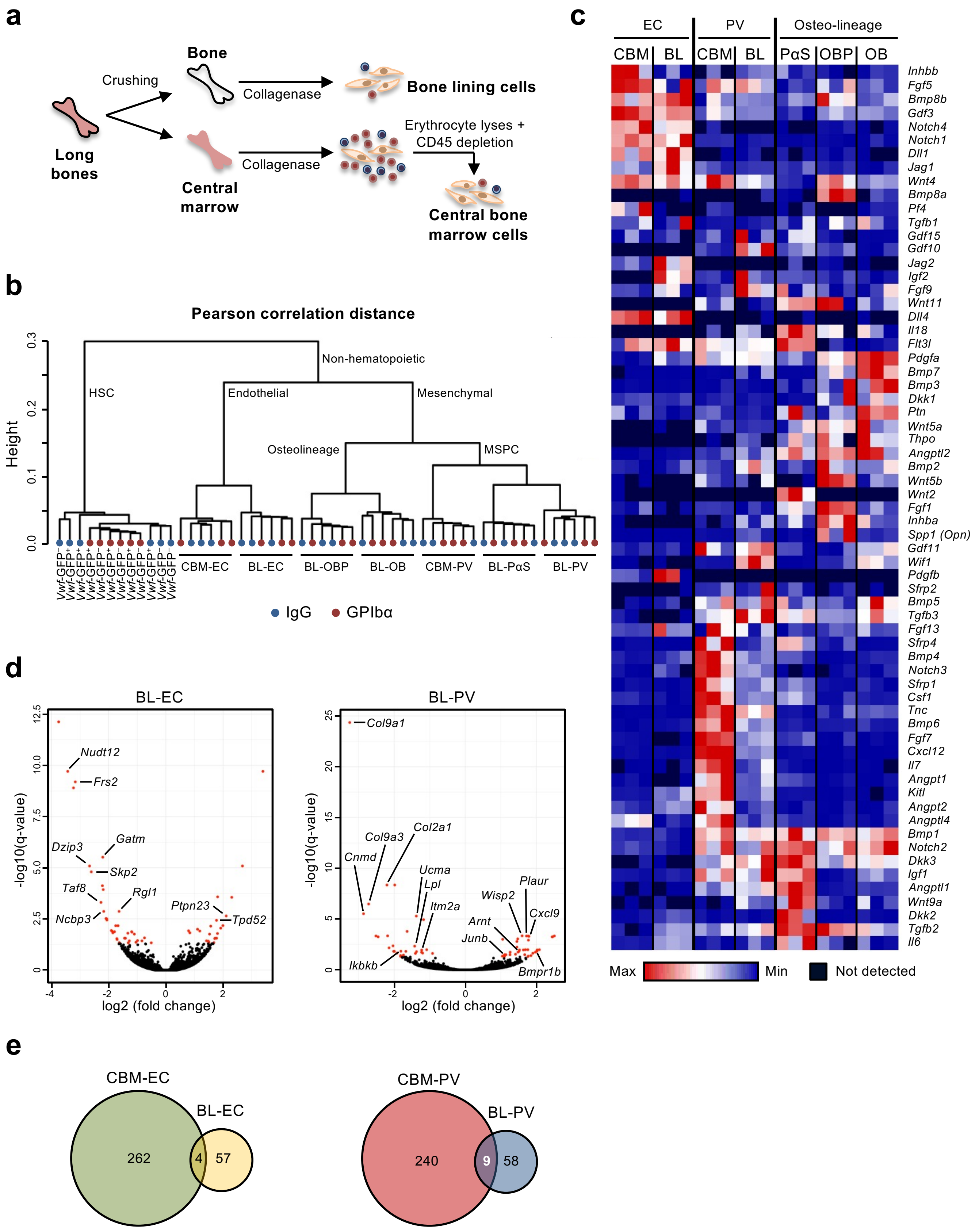

Supplementary Figure 3. RNA-sequencing analysis of bone marrow endothelial and stromal cells. (Related to main Figure 2)

(a) Illustration of procedure for analysis and isolation of niche cells from two different anatomic regions within BM, the bone lining (BL) and central bone marrow (CBM) regions, 1 day post treatment with anti-GPIbα or isotype (IgG) control antibody.

(b) Hierarchical clustering by cell population (one minus Pearson correlation) showing the relationship between the different cell populations analyzed, according to their global gene expression profile. EC, endothelial cells, PV, $LepR^{+}$ perivascular cells; OB, osteoblasts; OBP, osteoblast progenitors; PαS, $Pdfgr\alpha^{+}Sca1^{+}$ mesenchymal progenitors.

(c) Expression (FPKM) of different hematopoietic regulators in the different niche cell populations isolated from mice in homeostasis. Hierarchical clustering by gene (one minus Pearson correlation) was used to group genes with similar pattern of expression in the bone marrow niche.

(d) Volcano plots of genes differentially expressed in BL-EC and BL-PV cells isolated from mice in homeostasis and after platelet depletion. Red dots indicate significantly differentially expressed genes (adjusted *p* value (*q*)<0.05).

(e) Venn diagram showing number of genes differentially expressed exclusively or overlapping between CBM-EC and BL-EC (left) or CBM-PV and BL-PV (right) cells, isolated from mice in homeostasis or after platelet depletion.

Supplementary Figure 4

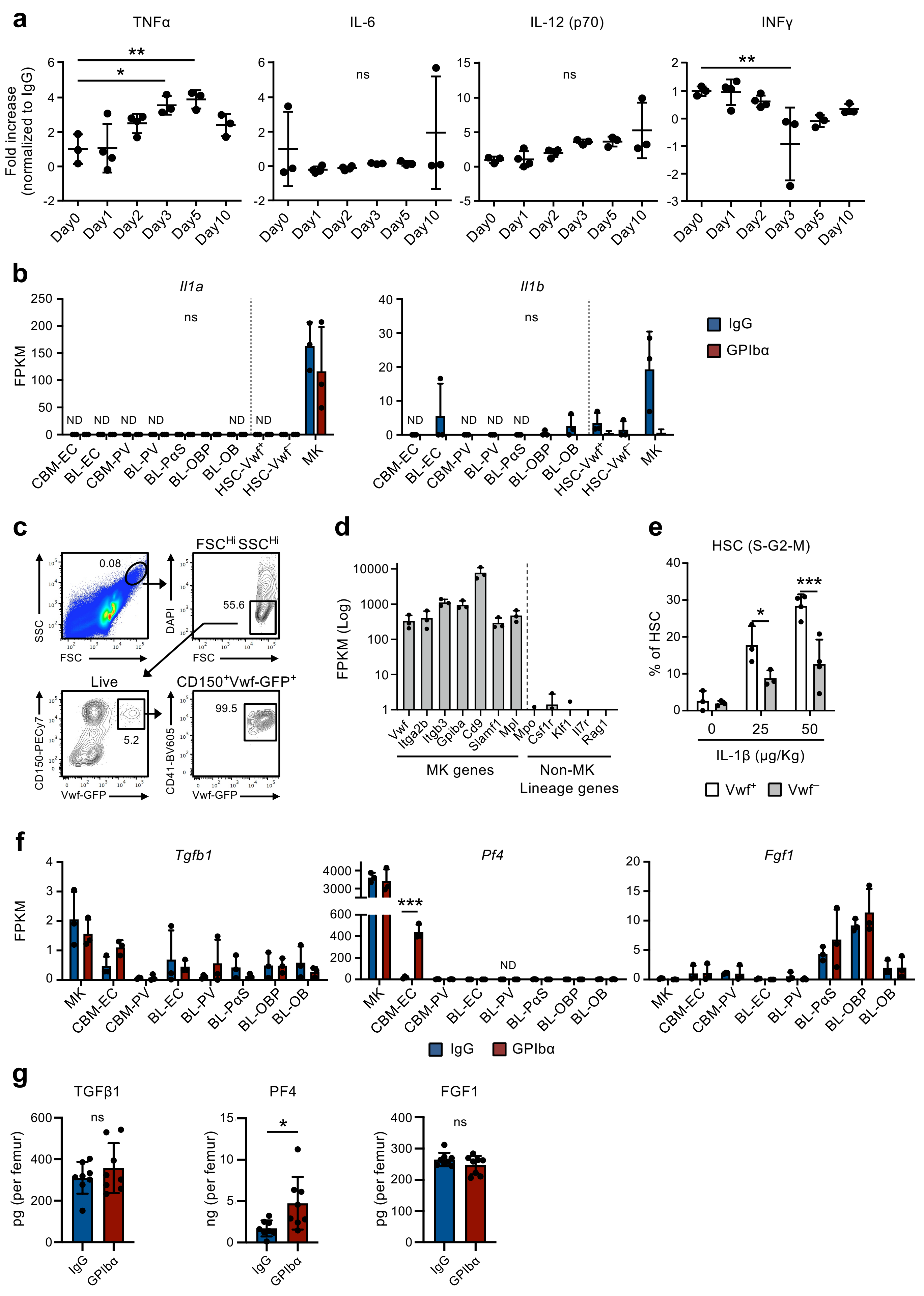

Supplementary Figure 4 - Expression of inflammatory cytokines in bone marrow.

(Related to main Figure 3)

(a) Mean ±SD levels (fold-increase relative to Day0) of the indicated cytokines in bone marrow extracellular fluid isolated from mice at the indicated time points post platelet depletion with GPIbα antibody. Control mice (Day 0) received isotype (IgG) control antibody. Data from 3 (Day0), 4 (Day1), 4 (Day2), 3 (Day3), 3 (Day5) and 3 (Day10) mice from 4 independent experiments. ***, $p<0.001$; **, $p<0.01$; *$p<0.05$; ns, non-significant (p>0.05); (1-way ANOVA with Dunnett's multiple comparisons).

(b) RNA-sequencing analysis of *Il1a* (left) and *Il1b* (right) genes expression (FPKM) in different niche cells, HSCs and Mks, isolated from the bone marrow of mice in homeostasis (IgG treated) or 1 day post platelet depletion (GPIbα treated). Data represent mean ±SD of 3 mice analyzed per condition. ND, not detected. ns, non-significant (p>0.05) between IgG and GPIbα, assessed with two-sided t-test.

(c) FACS analysis and gating strategy for sorting of bone marrow Mks. Mks were sorted as $FSC^{Hi}SSC^{Hi}DAPI^{-}CD150^{+}Vwf\text{-}GFP^{+}CD41^{+}$ cells. Numbers (represent percentage of parental gates

(d) Expression (FPKM) of Mk-associated genes and of non-Mk lineage-associated genes in the sorted cells. Data represent mean ±SD from 3 mice in 3 independent experiments.

(e) Cell cycle analysis of Vwf^{+} and Vwf^{-}HSCs from mice 1 day post intravenous administration of the indicated doses of IL-1β. Data are mean ±SD of 3 mice receiving 0 or 25 µg/Kg, and 4 mice receiving 50µg/Kg IL-1β, in 2 independent experiments. ***, $p<0.001$; *, $p<0.05$; 2-way ANOVA with Sidak's multiple comparisons).

(f) RNA-sequencing expression analysis of indicated niche-derived HSC regulators in niche cells, isolated from the bone marrow of mice in homeostasis (IgG treated) or 1 day post platelet depletion (GPIbα treated). Data represent mean ±SD of 3 mice analyzed per condition. ***, $p<0.001$ (two-sided t-Test); ND, not detected.

(g) Mean ±SD levels of the indicated soluble regulators in bone marrow extracellular fluid isolated from mice in homeostasis (IgG treated) or 1 day post platelet depletion (GPIbα treated). Data from 8 mice per condition. ***, $p<0.001$; *$p<0.05$; ns, non-significant, p>0.05; (two-sided t-Test).

Supplementary Figure 5

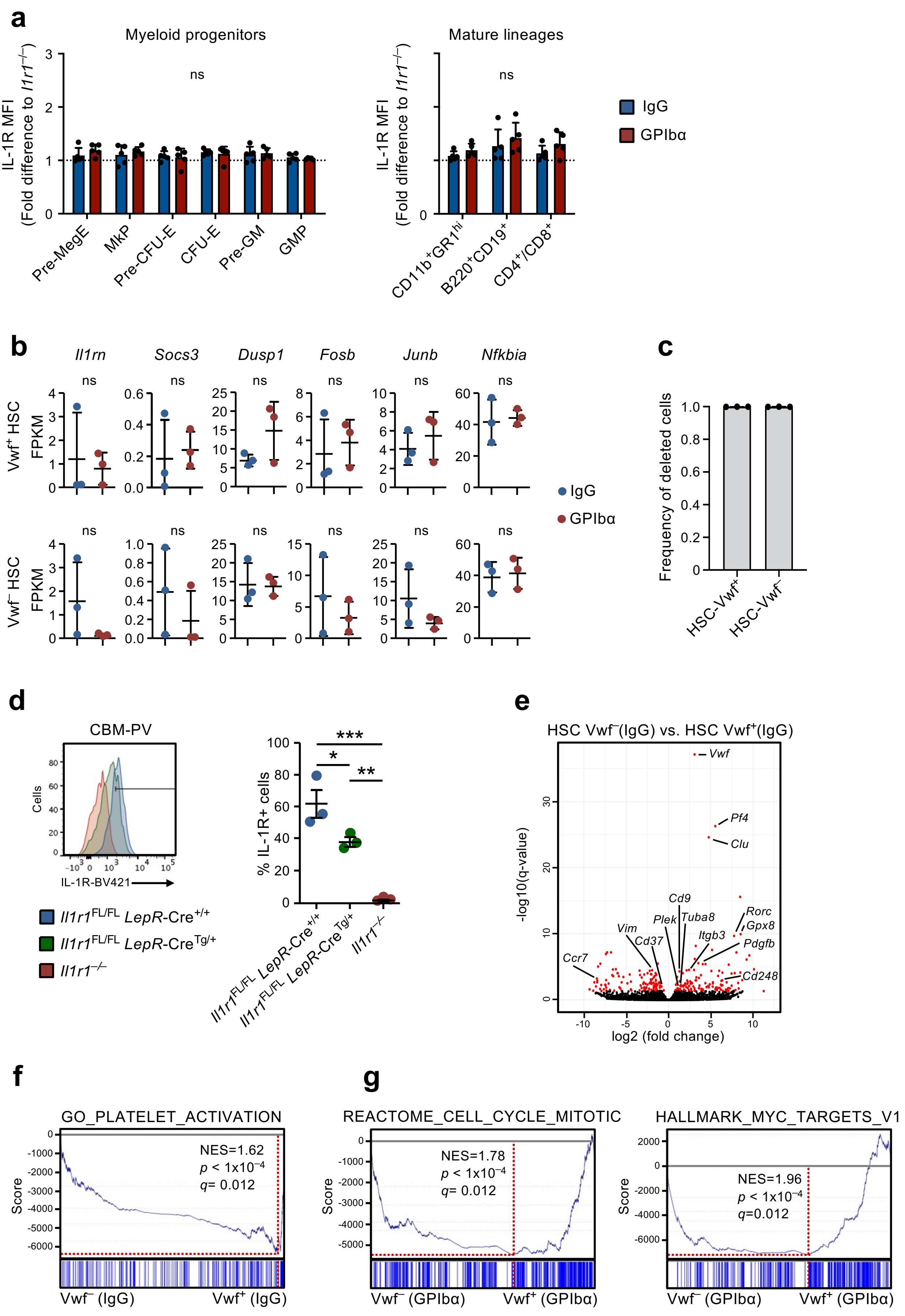

Supplementary Figure 5. RNA-sequencing analysis of Vwf^{+} and Vwf$^{–}$ HSCs after platelet depletion (Related to main Figures 4 and 5).

(a) Flow cytometric analysis of IL-1R expression in different hematopoietic progenitors and mature hematopoietic cells in the bone marrow of mice in homeostasis or 1 day post platelet depletion. Data represent the mean ±SD of MFI of each cell population, normalized for the MFI of the equivalent population in *Il1r1*$^{–/–}$ mice analyzed within the same experiments. Data are from 5 mice per condition in 2 independent experiments. ns, non-significant (p>0.05) between IgG and GPIbα, assessed with two-sided t-test.

(b) Expression of IL-1 signaling pathway affiliated genes in Vwf^{+} and Vwf$^{–}$ HSCs isolated from mice in homeostasis or 1 day post platelet depletion. Mean ±SD FPKM data of 3 biological replicates per condition. ns, non-significant (p>0.05) between IgG and GPIbα, assessed with two-sided t-test.

(c) Analysis of Vav-Cre-mediated deletion efficiency in HSCs of *Il1r1*$^{Fl/FL}$ at genomic DNA level by ddPCR. Data represent frequency of deleted Vwf^{+} or Vwf$^{–}$ HSCs isolated from *Il1r1*$^{FL/FL}$*Vav*-Cre$^{Tg/+}$ mice. Data from 3 mice, 3 FACS-sorted technical replicates from each mouse, each run as 2 ddPCR technical replicates.

(d) *Lepr*-Cre mediated deletion of the *Il1r1*^{FL} locus in LepR^{+} CBM-PV cells. Representative FACS analysis (left) and frequency of IL-1R^{+} cells in mice with the indicated genotypes. Data from 3 mice per genotype in 2 independent experiments. ***, p<0.001; **, p<0.01; *, p<0.05; 1-way ANOVA with Tukey's multiple comparisons).

(e) Volcano plot of Differentially expressed genes between Vwf^{+} and Vwf$^{–}$ HSCs in homeostasis (IgG) showing the Mk lineage priming of Vwf^{+} HSCs at molecular level. Red dots indicate significantly differentially expressed genes (adjusted p value (q)<0.05).

(f) Gene set enrichment analysis of the indicated platelet/Mk associated gene set.

(g) Gene set enrichment analysis of the indicated cell cycle associated gene sets comparing Vwf$^{–}$ and Vwf^{+} HSCs from GPIbα treated mice. NES, normalized enrichment score (or scaled score).

Supplementary Figure 6

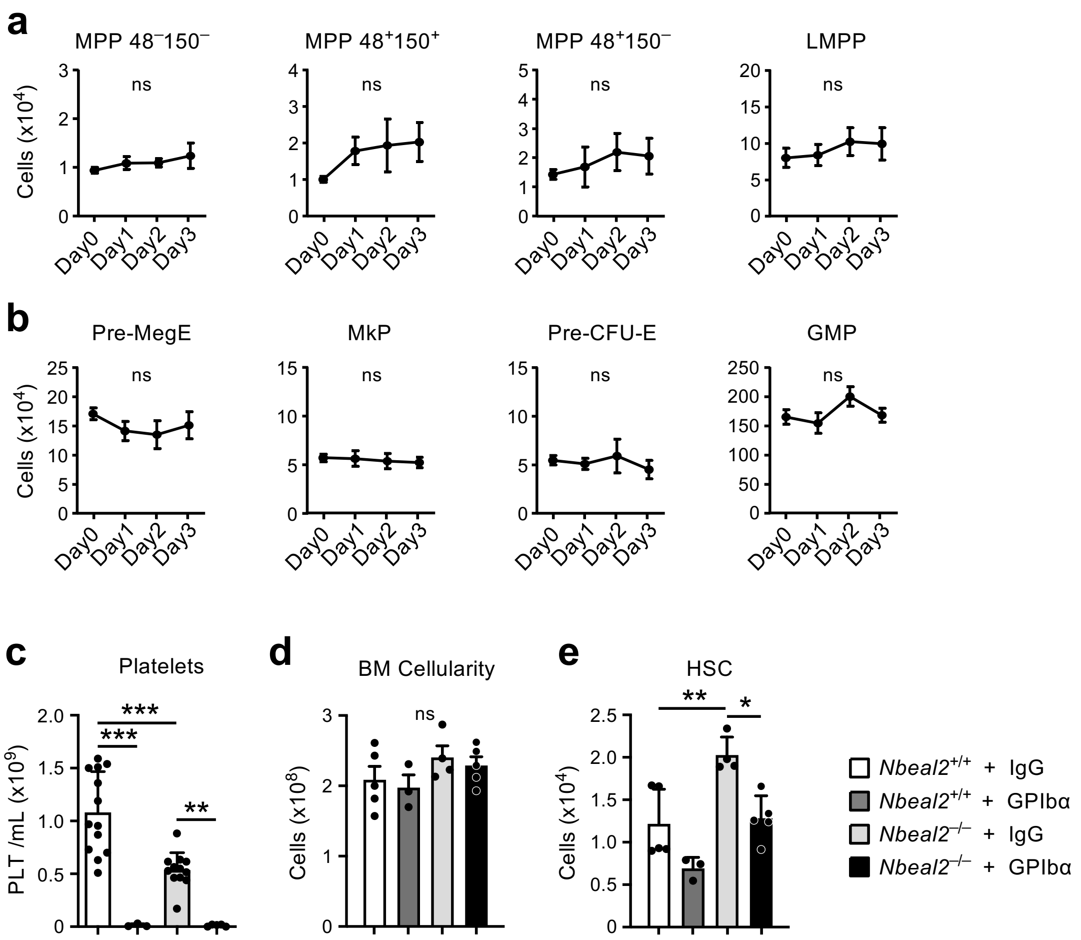

Supplementary Figure 6. Platelet activation is critical for the activation of Vwf⁺ HSCs in response to platelet depletion (Related to main Figure 6).

(a-b) Number of multipotent progenitors **(a)** and myeloid progenitors **(b)** at the indicated time points post platelet depletion with NEU. Data represent mean ±SEM of 5 (Day0), 4 (Day1), 5 (Day2) and 6 (Day3) mice from 4 independent experiments. No significant differences were observed for the absolute numbers of any cell population at the different time points.

(c-e) Peripheral blood platelet counts **(c)**, bone marrow cellularity **(d)** and absolute bone marrow HSC numbers **(e)** in *Nbeal2*$^{-/-}$ mice and littermate *Nbeal2*$^{+/+}$ controls in homeostasis and after platelet depletion with GPIbα antibody. **(c)** Data represent mean ±SD of 13 (Wt-IgG), 3 (Wt-GPIbα), 12 (*Nbeal2*$^{-/-}$-IgG) and 5 (*Nbeal2*$^{-/-}$-GPIbα) mice from 4 independent experiments. **(d-e)** Data represent mean ±SD of 5 (Wt-IgG), 3 (Wt-GPIbα), 4 (*Nbeal2*$^{-/-}$-IgG) and 5 (*Nbeal2*$^{-/-}$-GPIbα) mice from 3 independent experiments. For all panels, ****p*<0.001; ***p*<0.01; **p*<0.05; ns, non-significant (p>0.05), (1-way ANOVA with Tukey's multiple comparisons).

Supplementary Table 1

Antibody	Clone	Dilution	Supplier	Catalog #	Staining
Alcam-Bio	AAC06342	1/100	R&D Systems	FAB1172P	Niche cells
B220-PECF594	RA3-6B2	1/200	BD Biosciences	562313	Peripheral blood
B220-PECy5	RA3-6B2	1/400	BioLegend	103210	Lineage (HSPC, Myeloid progenitors)
B220-BUV395	RA3-6B2	1/200	BD Biosciences	563793	Lineage (HSPC)
CD105-Bio	MJ7/18	1/800	BioLegend	120404	Myeloid progenitors
CD105-BV421	MJ7/18	1/100	BD Biosciences	562760	Myeloid progenitors
CD11b/Mac1-AF700	M1/70	1/800	eBiosciences	56-0112-82	Mature cells
CD11b/Mac1-APC	M1/70	1/600	BioLegend	101212	Peripheral blood
CD140a/Pdfgra-APC	APA5	1/40	ebiosciences	17-1401-81	Niche cells
CD150-APC	TC15-12F12.2	1/100	BioLegend	115910	Myeloid progenitors, Peripheral blood
CD150-PECy7	TC15-12F12.2	1/400	BioLegend	115914	HSPC, HSC cell cycle, Platelets
CD150-BV785	TC15-12F12.2	1/200	BioLegend	115937	HSPC
CD16/32 (FcgR)-APC	93	1/600	eBiosciences	17-0161-81	Myeloid progenitors
CD16/32 (FcgR)-PE	93	1/400	eBiosciences	12-0161-81	Myeloid progenitors
CD19-PECy7	1D3	1/500	eBiosciences	25-0193-82	Mature cells in BM, Peripheral blood
CD31-PECy7	390	1/1600	eBiosciences	25-0311-82	Niche cells
CD41-APC	MWReg30	1/200	BioLegend	133914	Platelets
CD41-BV605	MWReg30	1/50	BioLegend	133921	Myeloid progenitors
CD41-PE	MWReg30	1/200	eBiosciences	12-0411-83	Peripheral blood
CD41-PECy7	MWReg30	1/800	eBiosciences	25-0411-82	Myeloid progenitors, Platelets
CD42b/GPIba-PE	Xia.G5	1/2.5	Emfret analytics	M040-2	HSPC, Myeloid progenitors
CD45.1-BV650	A20	1/25	BioLegend	110736	HSPC
CD45.1-PE	A20	1/100	eBiosciences	12-0453-83	Myeloid progenitors, Peripheral blood
CD45.2-AF700	104	1/100	BioLegend	109822	HSPC, Myeloid progenitors, Peripheral blood
CD45-AF700	30-F11	1/50	eBiosciences	56-0451-82	Niche cells
CD48-APC	HM48-1	1/600	BioLegend	103412	HSPC, HSC cell cycle
CD4-APCeF780	RM4-5	1/1200	eBiosciences	47-0042-82	Peripheral blood
CD4-PECy5	RM4-5	1/400	BioLegend	100514	Lineage (HSPC, Myeloid progenitors)
CD4-BUV395	RM4-5	1/400	BD Biosciences	740208	HSPC
CD5-PECy5	53-7.3	1/800	BioLegend	100610	Lineage (HSPC, Myeloid progenitors)
CD5-BUV395	53-7.3	1/100	BD Biosciences	740206	HSPC
CD62P-PE	Psel.KO2.3	1/100	eBiosciences	12-0626-80	Platelets
CD8-APCeF780	53-6.7	1/400	eBiosciences	47-0081-82	Peripheral blood
CD8a-PECy5	53-6.7	1/1200	BioLegend	100710	Lineage (HSPC, Myeloid progenitors)
CD8a-BUV395	53-6.7	1/200	BD Biosciences	563786	HSPC
cKit-APC-eF780	2B8	1/1200	eBiosciences	47-1171-82	HSPC, Myeloid progenitors, HSC cell cycle
Flt3-PE	A2F10	1/50	BioLegend	135306	HSPC staining
GR1-PECy5	RB6-8C5	1/800	BioLegend	108410	Lineage (HSC, Myeloid progenitors)
GR1-PO	RB6-8C5	1/100	Invitrogen	RM3030	Peripheral blood
GR1-BUV395	RB6-8C5	1/400	BD Biosciences	563849	HSPC
IL-1b-PE	166931	1/10	R&D Systems	IC4013P	Platelets
IL-1R-BV421	35F5	1/30	BD Biosciences	564387	Niche cells, HSPC, myloid progenitors, mature cells
Ki67-FITC	B56	1/50	BD Biosciences	556026	HSC cell cycle
Ki67-PE	B56	1/50	BD Biosciences	556027	HSC cell cycle
LepR-Bio	Polyclonal	1/100	R&D Systems	BAF497	Niche cells
NK1.1-PB	PK136	1/1200	BioLegend	108722	Peripheral blood
Rat IgG1k-BV421	R3-34	1/30	BD Biosciences	562868	Control for IL-1R Ab
Rat IgG2b-PE	141945	1/10	R&D Systems	IC013P	Control for IL1β Ab
Rat IgG-PE	Polyclonal	1/2.5	Emfret analytics	P-190-2	Control for CD42b Ab
SAv-BV421	-	1/200	BioLegend	405226	HSC-Biotin proliferation
SAv-PE	-	1/2000	BD Biosciences	12-4317-87	Niche cells
SAv-PETxR	-	1/800	BD Biosciences	551487	Myeloid progenitors
Sca1-BV605	D7	1/150	BioLegend	405229	HSPC and Myeloid progenitors
Sca1-FITC	E13-161.7	1/200	BioLegend	122506	HSC cell cycle
Sca1-PerCP-Cy5.5	D7	1/300	eBiosciences	45-5981-80	HSC cell cycle
Sca1-PECy7	E13-161.7	1/400	BioLegend	122514	HSPC
Ter119-PECy5	TER-119	1600	BioLegend	116210	Lineage (HSPC), Niche cells, Peripheral blood
Ter119-BUV395	TER-119	1/100	BD Biosciences	563827	HSPC

Supplementary Table 1 – Flow Cytometry antibodies and application
