# Supplementary material for: Perivascular niche cells sense thrombocytopenia and activate hematopoietic stem cells in an IL-1 dependent manner
Source: Nat Commun. 2023 Sep 28;14:6062. doi: 10.1038/s41467-023-41691-y (PMC10539537; doi:10.1038/s41467-023-41691-y)
Supplement: Supplementary file 3 — Description of additional supplementary files [file 41467_2023_41691_MOESM3_ESM.pdf]

Description of Additional Supplementary Files document

Supplementary Data 1 - Expression of known genes characterizing different niche cell populations and h ematopoietic regulators

Supplementary Data 2 - Differentially expressed genes between IgG and GPIba CBM-EC and BL-EC cells

Supplementary Data 3 - Differentially expressed genes between IgG and GPIba CBM-PV and BL-PV cells

Supplementary Data 4 - Differentially expressed genes between IgG and GPIba BL-PαS, BLOBP and BL-OB cells

Supplementary Data 5 - Gene ontology analysis on genes differentially expressed in CBM-PV and CBM-EC

Supplementary Data 6 - Differentially expressed genes between IgG and GPIba Vwf+ HSCs

Supplementary Data 7 - Differentially expressed genes between IgG and GPIba Vwf- HSCs

Supplementary Data 8 - Gene ontology analysis on genes differentially expressed only in VWF+ HSCs
