# Supplementary material for: Perivascular niche cells sense thrombocytopenia and activate hematopoietic stem cells in an IL-1 dependent manner
Source: Nat Commun. 2023 Sep 28;14:6062. doi: 10.1038/s41467-023-41691-y (PMC10539537; doi:10.1038/s41467-023-41691-y)
Supplement: Supplementary file 12 — Reporting Summary [file 41467_2023_41691_MOESM12_ESM.pdf]

Corresponding author(s): Tiago C. Luis, Sten Eirik W. Jacobsen

Last updated by author(s): September 1st, 2023

Reporting Summary

Nature Portfolio wishes to improve the reproducibility of the work that we publish. This form provides structure for consistency and transparency in reporting. For further information on Nature Portfolio policies, see our Editorial Policies and the Editorial Policy Checklist.

Statistics

For all statistical analyses, confirm that the following items are present in the figure legend, table legend, main text, or Methods section.

n/a	Confirmed	
☐	☒	The exact sample size (*n*) for each experimental group/condition, given as a discrete number and unit of measurement
☐	☒	A statement on whether measurements were taken from distinct samples or whether the same sample was measured repeatedly
☐	☒	The statistical test(s) used AND whether they are one- or two-sided<br>*Only common tests should be described solely by name; describe more complex techniques in the Methods section.*
☐	☒	A description of all covariates tested
☐	☒	A description of any assumptions or corrections, such as tests of normality and adjustment for multiple comparisons
☐	☒	A full description of the statistical parameters including central tendency (e.g. means) or other basic estimates (e.g. regression coefficient) AND variation (e.g. standard deviation) or associated estimates of uncertainty (e.g. confidence intervals)
☐	☒	For null hypothesis testing, the test statistic (e.g. *F*, *t*, *r*) with confidence intervals, effect sizes, degrees of freedom and *P* value noted<br>*Give P values as exact values whenever suitable.*
☒	☐	For Bayesian analysis, information on the choice of priors and Markov chain Monte Carlo settings
☒	☐	For hierarchical and complex designs, identification of the appropriate level for tests and full reporting of outcomes
☐	☒	Estimates of effect sizes (e.g. Cohen's *d*, Pearson's *r*), indicating how they were calculated

Our web collection on statistics for biologists contains articles on many of the points above.

Software and code

Policy information about availability of computer code

Data collection	Flow cytometry analysis was performed on a BD LSRII and a BD Fortessa X20 (BD Biosciences). Cell sorting was performed on a BD FACS Ariallu, AriaIII and Fusion (BD Biosciences). Immunofluorescence images were acquired with a Zeiss 780 LSM multi-photon/confocal upright microscope equipped with a MaiTai laser. ddPCR was performed on a QX200 droplet generator and a QX200 droplet reader (Bio-Rad). RNA sequencing samples were sequenced on a HiSeq2500 and HiSeq4000 (Illumina). Blood parameters were analyzed using ABX PENTRA 60C+ (Horiba) or XP-300 (Sysmex) automated blood cell analyzers.
Data analysis	Flow cytometry data analysis was performed on FlowJo software (TreeStar Inc/BD Biosciences). Immunofluorescence image analysis was performed with Imaris 7.5.0 software (Bitplane). ddPCR results were analysed using QuantaSoft v1.5.38.1118 software (Bio-Rad).<br>For RNA sequencing analysis adapter removal was performed with Trimgalore (v.1.2.1, https://github.com/FelixKrueger/TrimGalore), alignment was performed to the mm10 mouse built with refSeq transcriptome annotation using tophat (version 2.0.10). Reads were sorted using samtools (version 0.1.19), only primary alignments were used for subsequent steps. Read counting to transcripts was performed using the packages Rsamtools (version 1.18.2) (http://bioconductor.org/packages/release/bioc/html/Rsamtools.html) and GenomicAlignments (version 1.2.1) in R version (3.1.1). FPKM was calculated using the DESeq2 fpkm function. Differential expression analysis was performed using DESeq2 (version 1.18.1) independently for each comparison. Principal component analysis was performed using the prcomp R function. GSEA analysis was performed against datasets from msigdb converted to mouse identifiers available from (http://bioinf.wehi.edu.au/software/MSigDB/) using liger (https://github.com/JEFworks/liger). GO Analysis was performed using the GOStats R package (version 2.44.0). Hierarchical clustering was generated based on Pearson correlation distance using the R function hclust and the 'ward.D2' method on the rlog transformed count values (calculated with DESeq2). Heatmaps were generated with Morpheus (Broad Institute). Venn diagrams were performed with BioVenn software. Figure preparation was performed with the dendextend R package (version 1.8.0). Code used in this manuscript has been deposited in Zenodo (https://zenodo.org/record/8283315).

For manuscripts utilizing custom algorithms or software that are central to the research but not yet described in published literature, software must be made available to editors and reviewers. We strongly encourage code deposition in a community repository (e.g. GitHub). See the Nature Portfolio guidelines for submitting code & software for further information.

Data

Policy information about availability of data

All manuscripts must include a data availability statement. This statement should provide the following information, where applicable:
- Accession codes, unique identifiers, or web links for publicly available datasets
- A description of any restrictions on data availability
- For clinical datasets or third party data, please ensure that the statement adheres to our policy

The RNA-Sequencing data generated in this study have been deposited in NCBI Gene Expression Omnibus (GEO) and are accessible through GSE121249 (NCBI tracking system #19505382). GSEA analysis was performed against datasets from msigdb available from (http://bioinf.wehi.edu.au/software/MSigDB/). The source data for Figs.1-6 and Supplementary Figs.1-2, 4-6 are provided as a Source Data file. All other data that supports the findings of this study are available from the corresponding authors upon request.

Research involving human participants, their data, or biological material

Policy information about studies with human participants or human data. See also policy information about sex, gender (identity/presentation), and sexual orientation and race, ethnicity and racism.

Reporting on sex and gender	*Use the terms sex (biological attribute) and gender (shaped by social and cultural circumstances) carefully in order to avoid confusing both terms. Indicate if findings apply to only one sex or gender; describe whether sex and gender were considered in study design; whether sex and/or gender was determined based on self-reporting or assigned and methods used. Provide in the source data disaggregated sex and gender data, where this information has been collected, and if consent has been obtained for sharing of individual-level data; provide overall numbers in this Reporting Summary. Please state if this information has not been collected. Report sex- and gender-based analyses where performed, justify reasons for lack of sex- and gender-based analysis.*
Reporting on race, ethnicity, or other socially relevant groupings	*Please specify the socially constructed or socially relevant categorization variable(s) used in your manuscript and explain why they were used. Please note that such variables should not be used as proxies for other socially constructed/relevant variables (for example, race or ethnicity should not be used as a proxy for socioeconomic status). Provide clear definitions of the relevant terms used, how they were provided (by the participants/respondents, the researchers, or third parties), and the method(s) used to classify people into the different categories (e.g. self-report, census or administrative data, social media data, etc.) Please provide details about how you controlled for confounding variables in your analyses.*
Population characteristics	*Describe the covariate-relevant population characteristics of the human research participants (e.g. age, genotypic information, past and current diagnosis and treatment categories). If you filled out the behavioural & social sciences study design questions and have nothing to add here, write "See above."*
Recruitment	*Describe how participants were recruited. Outline any potential self-selection bias or other biases that may be present and how these are likely to impact results.*
Ethics oversight	*Identify the organization(s) that approved the study protocol.*

Note that full information on the approval of the study protocol must also be provided in the manuscript.

Field-specific reporting

Please select the one below that is the best fit for your research. If you are not sure, read the appropriate sections before making your selection.

☒ Life sciences ☐ Behavioural & social sciences ☐ Ecological, evolutionary & environmental sciences

For a reference copy of the document with all sections, see nature.com/documents/nr-reporting-summary-flat.pdf

Life sciences study design

All studies must disclose on these points even when the disclosure is negative.

Sample size	Sample sizes are indicated in all figure legends. No Statistical methods were used to predetermine the experimental sample size. Sample sizes were chosen based on previous experience and published studies performing similar types of experiments.
Data exclusions	No data was excluded
Replication	Multiple independent experiments were performed for all data sets. Numbers of biological replicates, technical replicates and experiments performed are indicated in figure legends for all data sets.

Randomization	Statistical methods for randomization were not applied. This study is based on transgenic mouse models that need to be genotyped before being allocated to experiments.
Blinding	Blinding was not performed given that mice need to be genotyped to allocate them to the different experimental groups.

Behavioural & social sciences study design

All studies must disclose on these points even when the disclosure is negative.

Study description	*Briefly describe the study type including whether data are quantitative, qualitative, or mixed-methods (e.g. qualitative cross-sectional, quantitative experimental, mixed-methods case study).*
Research sample	*State the research sample (e.g. Harvard university undergraduates, villagers in rural India) and provide relevant demographic information (e.g. age, sex) and indicate whether the sample is representative. Provide a rationale for the study sample chosen. For studies involving existing datasets, please describe the dataset and source.*
Sampling strategy	*Describe the sampling procedure (e.g. random, snowball, stratified, convenience). Describe the statistical methods that were used to predetermine sample size OR if no sample-size calculation was performed, describe how sample sizes were chosen and provide a rationale for why these sample sizes are sufficient. For qualitative data, please indicate whether data saturation was considered, and what criteria were used to decide that no further sampling was needed.*
Data collection	*Provide details about the data collection procedure, including the instruments or devices used to record the data (e.g. pen and paper, computer, eye tracker, video or audio equipment) whether anyone was present besides the participant(s) and the researcher, and whether the researcher was blind to experimental condition and/or the study hypothesis during data collection.*
Timing	*Indicate the start and stop dates of data collection. If there is a gap between collection periods, state the dates for each sample cohort.*
Data exclusions	*If no data were excluded from the analyses, state so OR if data were excluded, provide the exact number of exclusions and the rationale behind them, indicating whether exclusion criteria were pre-established.*
Non-participation	*State how many participants dropped out/declined participation and the reason(s) given OR provide response rate OR state that no participants dropped out/declined participation.*
Randomization	*If participants were not allocated into experimental groups, state so OR describe how participants were allocated to groups, and if allocation was not random, describe how covariates were controlled.*

Ecological, evolutionary & environmental sciences study design

All studies must disclose on these points even when the disclosure is negative.

Study description	*Briefly describe the study. For quantitative data include treatment factors and interactions, design structure (e.g. factorial, nested, hierarchical), nature and number of experimental units and replicates.*
Research sample	*Describe the research sample (e.g. a group of tagged Passer domesticus, all Stenocereus thurberi within Organ Pipe Cactus National Monument), and provide a rationale for the sample choice. When relevant, describe the organism taxa, source, sex, age range and any manipulations. State what population the sample is meant to represent when applicable. For studies involving existing datasets, describe the data and its source.*
Sampling strategy	*Note the sampling procedure. Describe the statistical methods that were used to predetermine sample size OR if no sample-size calculation was performed, describe how sample sizes were chosen and provide a rationale for why these sample sizes are sufficient.*
Data collection	*Describe the data collection procedure, including who recorded the data and how.*
Timing and spatial scale	*Indicate the start and stop dates of data collection, noting the frequency and periodicity of sampling and providing a rationale for these choices. If there is a gap between collection periods, state the dates for each sample cohort. Specify the spatial scale from which the data are taken*
Data exclusions	*If no data were excluded from the analyses, state so OR if data were excluded, describe the exclusions and the rationale behind them, indicating whether exclusion criteria were pre-established.*
Reproducibility	*Describe the measures taken to verify the reproducibility of experimental findings. For each experiment, note whether any attempts to repeat the experiment failed OR state that all attempts to repeat the experiment were successful.*
Randomization	*Describe how samples/organisms/participants were allocated into groups. If allocation was not random, describe how covariates were controlled. If this is not relevant to your study, explain why.*
Blinding	*Describe the extent of blinding used during data acquisition and analysis. If blinding was not possible, describe why OR explain why blinding was not relevant to your study.*

Did the study involve field work? ☐ Yes ☐ No

Field work, collection and transport

Field conditions	*Describe the study conditions for field work, providing relevant parameters (e.g. temperature, rainfall).*
Location	*State the location of the sampling or experiment, providing relevant parameters (e.g. latitude and longitude, elevation, water depth).*
Access & import/export	*Describe the efforts you have made to access habitats and to collect and import/export your samples in a responsible manner and in compliance with local, national and international laws, noting any permits that were obtained (give the name of the issuing authority, the date of issue, and any identifying information).*
Disturbance	*Describe any disturbance caused by the study and how it was minimized.*

Reporting for specific materials, systems and methods

We require information from authors about some types of materials, experimental systems and methods used in many studies. Here, indicate whether each material, system or method listed is relevant to your study. If you are not sure if a list item applies to your research, read the appropriate section before selecting a response.

Materials & experimental systems

n/a	Involved in the study	
☐	☒	Antibodies
☒	☐	Eukaryotic cell lines
☒	☐	Palaeontology and archaeology
☐	☒	Animals and other organisms
☒	☐	Clinical data
☒	☐	Dual use research of concern
☒	☐	Plants

Methods

n/a	Involved in the study	
☒	☐	ChIP-seq
☐	☒	Flow cytometry
☒	☐	MRI-based neuroimaging

Antibodies

Antibodies used

Flow Cytometry antibodies (Also summarized in Supplementary Table 1 of the manuscript):
Alcam-Bio (clone AAC06342; R&D Systems; Cat# FAB1172P),
B220-PECF594 (clone RA3-6B2; BD Biosciences; Cat# 562313),
B220-PECy5 (clone RA3-6B2; BioLegend; Cat# 103210),
B220-BUV395 (clone RA3-6B2; BD Biosciences; Cat# 563793),
CD105-Bio (clone MJ7/18; BioLegend; Cat# 120404),
CD105-BV421 (clone MJ7/18; BD Biosciences; Cat# 562760),
CD11b/Mac1-AF700 (clone M1/70; eBiosciences; Cat# 56-0112-82),
CD11b/Mac1-APC (clone M1/70; BioLegend; Cat# 101212),
CD140a/Pdfgra-APC (clone APA5; ebiosciences; Cat# 17-1401-81),
CD150-APC (clone TC15-12F12.2; BioLegend; Cat# 115910),
CD150-PECy7 (clone TC15-12F12.2; BioLegend; Cat# 115914),
CD150-BV785 (clone TC15-12F12.2; BioLegend; Cat# 115937),
CD16/32 (FcgR)-APC (clone 93; eBiosciences; Cat# 17-0161-81),
CD16/32 (FcgR)-PE (clone 93; eBiosciences; Cat# 12-0161-81),
CD19-PECy7 (clone 1D3; eBiosciences; Cat# 25-0193-82),
CD31-PECy7 (clone 390; eBiosciences; Cat# 25-0311-82),
CD41-APC (clone MWReg30; BioLegend; Cat# 133914),
CD41-BV605 (clone MWReg30; BioLegend; Cat# 133921),
CD41-PE (clone MWReg30; eBiosciences; Cat# 12-0411-83),
CD41-PECy7 (clone MWReg30; eBiosciences; Cat# 25-0411-82),
CD42b/GPIba-PE (clone Xia.G5 ; Emfret Analytics; Cat# M040-2),
CD45.1-BV650 (clone A20; BioLegend; Cat# 110736),
CD45.1-PE (clone A20; eBiosciences; Cat# 12-0453-83),
CD45.2-AF700 (clone 104; BioLegend; Cat# 109822),
CD45-AF700 (clone 30-F11; eBiosciences; Cat# 56-0451-82),
CD48-APC (clone HM48-1; BioLegend; Cat# 103412),
CD4-APCeF780 (clone RM4-5; eBiosciences; Cat# 47-0042-82),
CD4-PECy5 (clone RM4-5; BioLegend; Cat# 100514),
CD4-BUV395 (clone RM4-5; BD Biosciences; Cat# 740208),
CD5-PECy5 (clone 53-7.3; BioLegend; Cat# 100610),
CD5-BUV395 (clone 53-7.3; BD Biosciences; Cat# 740206),

CD62P-PE (clone Psel.KO2.3; eBiosciences; Cat# 12-0626-80),
CD8-APCeF780 (clone 53-6.7; eBiosciences; Cat# 47-0081-82),
CD8a-PECy5 (clone 53-6.7; BioLegend; Cat# 100710),
CD8a-BUV395 (clone 53-6.7; BD Biosciences; Cat# 563786),
cKit-APC-eF780 (clone 2B8; eBiosciences; Cat# 47-1171-82),
Flt3-PE (clone A2F10 ;BioLegend; Cat# 135306),
GR1-PECy5 (clone RB6-8C5; BioLegend; Cat# 108410),
GR1-PO (clone RB6-8C5; Invitrogen; Cat# RM3030),
GR1-BUV395 (clone RB6-8C5; BD Biosciences; Cat# 563849),
IL-1b-PE (clone 166931; R&D Systems; Cat# IC4013P),
IL-1R-BV421 (clone 35F5; BD Biosciences; Cat# 564387),
Ki67-FITC (clone B56; BD Biosciences; Cat# 556026),
Ki67-PE (clone B56; BD Biosciences; Cat# 556027),
LepR-Bio (Polyclonal; R&D Systems; Cat# BAF497),
NK1.1-PB (clone PK136; BioLegend; Cat# 108722),
Rat IgG1k-BV421 (clone R3-34; BD Biosciences; Cat# 562868),
Rat IgG2b-PE (clone 141945; R&D Systems; Cat# IC013P),
Rat IgG-PE (Polyclonal; Emfret Analytics; Cat# P-190-2),
SAv-BV421 (BioLegend; Cat# 405226),
SAv-PE (BD Biosciences; Cat# 12-4317-87),
SAv-PETxR (BD Biosciences; Cat# 551487),
Sca1-BV605 (clone D7; BioLegend; Cat# 405229),
Sca1-FITC (clone E13-161.7; BioLegend; Cat# 122506),
Sca1-PerCP-Cy5.5 (clone D7; eBiosciences; Cat# 45-5981-80),
Sca1-PECy7 (clone E13-161.7; BioLegend; Cat# 122514),
Ter119-PECy5 (clone TER-119; BioLegend; Cat# 116210),
Ter119-BUV395 (clone TER-119; BD Biosciences; Cat# 563827).

Imaging antibodies:
CD31-AF647 (clone Mec13.3; BioLegend; Cat# 102516),
CD144-AF647 (clone BV13; BioLegend; Cat# 138006)
CD41-Bio (clone eBioMwreg30; eBiosciences; Cat# 13-0411-82),
CD150-PE (clone TC15-12F12.2; BioLegend; Cat# 115904),
SAv-eF450 (BioLegend; Cat# 405226).

Platelet depletion Antibodies:
CD42b (R300; Emfret Analytics; Cat# R300),
Rat IgG (C301; Emfret Analytics; Cat# C301),
Nit E (Provided by Prof Heyu Ni).

Validation

Antibodies used in this study are commercially available and have been validated in previous publications (Luis et al, Nature Immunology, 2016; Sanjuan-Pla et al, Nature, 2013; Carrelha et al, Nature, 2018; Li et al, Nature Communications, 2015). All flow cytometry antibodies are titrated for the specific application they were used, FMO controls were used in all experiments, and Isotype controls used where appropriate, as described in the methods section. IL-1R-BV421 antibody was further validated in this study by staining cells from Il1r1-/- mice, which were used as negative control.

Eukaryotic cell lines

Policy information about cell lines and Sex and Gender in Research

Cell line source(s)

State the source of each cell line used and the sex of all primary cell lines and cells derived from human participants or vertebrate models.

Authentication

Describe the authentication procedures for each cell line used OR declare that none of the cell lines used were authenticated.

Mycoplasma contamination

Confirm that all cell lines tested negative for mycoplasma contamination OR describe the results of the testing for mycoplasma contamination OR declare that the cell lines were not tested for mycoplasma contamination.

Commonly misidentified lines
(See ICLAC register)

Name any commonly misidentified cell lines used in the study and provide a rationale for their use.

Palaeontology and Archaeology

Specimen provenance

Provide provenance information for specimens and describe permits that were obtained for the work (including the name of the issuing authority, the date of issue, and any identifying information). Permits should encompass collection and, where applicable, export.

Specimen deposition

Indicate where the specimens have been deposited to permit free access by other researchers.

Dating methods

If new dates are provided, describe how they were obtained (e.g. collection, storage, sample pretreatment and measurement), where they were obtained (i.e. lab name), the calibration program and the protocol for quality assurance OR state that no new dates are

provided.

☐ Tick this box to confirm that the raw and calibrated dates are available in the paper or in Supplementary Information.

Ethics oversight	*Identify the organization(s) that approved or provided guidance on the study protocol, OR state that no ethical approval or guidance was required and explain why not.*

Note that full information on the approval of the study protocol must also be provided in the manuscript.

Animals and other research organisms

Policy information about studies involving animals; ARRIVE guidelines recommended for reporting animal research, and Sex and Gender in Research

Laboratory animals	This study uses mice of the following strains: Vwf-GFP, Vwf-TdTomato, Osx-GFP::Cre, Il1r1–/–, Il1r1Fl/Fl mice, Vav-Cre, LepR-Cre and Nbeal2–/–. All mouse lines were back-crossed for at least 6 generations onto a C57BL/6J genetic background and litter-mate controls were used in all experiments. Mice used in this study were between 7 and 14 weeks old
Wild animals	No wild animals wre used in this study.
Reporting on sex	Mice of both sexes were used in this study.
Field-collected samples	No field-collected samples were used in this study.
Ethics oversight	All mice were bred and maintained in accordance with UK Home Office regulations. All procedures were performed under project licenses 30/3103 and P2FF90EE8 approved by the Oxford University Clinical Medicine Ethical Review Committee.

Note that full information on the approval of the study protocol must also be provided in the manuscript.

Clinical data

Policy information about clinical studies
All manuscripts should comply with the ICMJE guidelines for publication of clinical research and a completed CONSORT checklist must be included with all submissions.

Clinical trial registration	*Provide the trial registration number from ClinicalTrials.gov or an equivalent agency.*
Study protocol	*Note where the full trial protocol can be accessed OR if not available, explain why.*
Data collection	*Describe the settings and locales of data collection, noting the time periods of recruitment and data collection.*
Outcomes	*Describe how you pre-defined primary and secondary outcome measures and how you assessed these measures.*

Dual use research of concern

Policy information about dual use research of concern

Hazards

Could the accidental, deliberate or reckless misuse of agents or technologies generated in the work, or the application of information presented in the manuscript, pose a threat to:

No	Yes	
☐	☐	Public health
☐	☐	National security
☐	☐	Crops and/or livestock
☐	☐	Ecosystems
☐	☐	Any other significant area

Experiments of concern

Does the work involve any of these experiments of concern:

No	Yes	
☐	☐	Demonstrate how to render a vaccine ineffective
☐	☐	Confer resistance to therapeutically useful antibiotics or antiviral agents
☐	☐	Enhance the virulence of a pathogen or render a nonpathogen virulent
☐	☐	Increase transmissibility of a pathogen
☐	☐	Alter the host range of a pathogen
☐	☐	Enable evasion of diagnostic/detection modalities
☐	☐	Enable the weaponization of a biological agent or toxin
☐	☐	Any other potentially harmful combination of experiments and agents

Plants

Seed stocks	*Report on the source of all seed stocks or other plant material used. If applicable, state the seed stock centre and catalogue number. If plant specimens were collected from the field, describe the collection location, date and sampling procedures.*
Novel plant genotypes	*Describe the methods by which all novel plant genotypes were produced. This includes those generated by transgenic approaches, gene editing, chemical/radiation-based mutagenesis and hybridization. For transgenic lines, describe the transformation method, the number of independent lines analyzed and the generation upon which experiments were performed. For gene-edited lines, describe the editor used, the endogenous sequence targeted for editing, the targeting guide RNA sequence (if applicable) and how the editor was applied.*
Authentication	*Describe any authentication procedures for each seed stock used or novel genotype generated. Describe any experiments used to assess the effect of a mutation and, where applicable, how potential secondary effects (e.g. second site T-DNA insertions, mosiacism, off-target gene editing) were examined.*

ChIP-seq

Data deposition

☐ Confirm that both raw and final processed data have been deposited in a public database such as GEO.

☐ Confirm that you have deposited or provided access to graph files (e.g. BED files) for the called peaks.

Data access links<br>*May remain private before publication.*	*For "Initial submission" or "Revised version" documents, provide reviewer access links. For your "Final submission" document, provide a link to the deposited data.*
Files in database submission	*Provide a list of all files available in the database submission.*
Genome browser session<br>(e.g. UCSC)	*Provide a link to an anonymized genome browser session for "Initial submission" and "Revised version" documents only, to enable peer review. Write "no longer applicable" for "Final submission" documents.*

Methodology

Replicates	*Describe the experimental replicates, specifying number, type and replicate agreement.*
Sequencing depth	*Describe the sequencing depth for each experiment, providing the total number of reads, uniquely mapped reads, length of reads and whether they were paired- or single-end.*
Antibodies	*Describe the antibodies used for the ChIP-seq experiments; as applicable, provide supplier name, catalog number, clone name, and lot number.*
Peak calling parameters	*Specify the command line program and parameters used for read mapping and peak calling, including the ChIP, control and index files used.*
Data quality	*Describe the methods used to ensure data quality in full detail, including how many peaks are at FDR 5% and above 5-fold enrichment.*
Software	*Describe the software used to collect and analyze the ChIP-seq data. For custom code that has been deposited into a community repository, provide accession details.*

Flow Cytometry

Plots

Confirm that:

☒ The axis labels state the marker and fluorochrome used (e.g. CD4-FITC).

☒ The axis scales are clearly visible. Include numbers along axes only for bottom left plot of group (a 'group' is an analysis of identical markers).

☒ All plots are contour plots with outliers or pseudocolor plots.

☒ A numerical value for number of cells or percentage (with statistics) is provided.

Methodology

Sample preparation	See methods section.
Instrument	LSRII, BD Fortessa X20, BD FACS Ariallu, AriaIII and Fusion (all from BD Biosciences).
Software	Diva, FlowJo.
Cell population abundance	Variable.
Gating strategy	Shown in figures and described in main text.

☒ Tick this box to confirm that a figure exemplifying the gating strategy is provided in the Supplementary Information.

Magnetic resonance imaging

Experimental design

Design type	*Indicate task or resting state; event-related or block design.*
Design specifications	*Specify the number of blocks, trials or experimental units per session and/or subject, and specify the length of each trial or block (if trials are blocked) and interval between trials.*
Behavioral performance measures	*State number and/or type of variables recorded (e.g. correct button press, response time) and what statistics were used to establish that the subjects were performing the task as expected (e.g. mean, range, and/or standard deviation across subjects).*

Acquisition

Imaging type(s)	*Specify: functional, structural, diffusion, perfusion.*
Field strength	*Specify in Tesla*
Sequence & imaging parameters	*Specify the pulse sequence type (gradient echo, spin echo, etc.), imaging type (EPI, spiral, etc.), field of view, matrix size, slice thickness, orientation and TE/TR/flip angle.*
Area of acquisition	*State whether a whole brain scan was used OR define the area of acquisition, describing how the region was determined.*

Diffusion MRI ☐ Used ☐ Not used

Preprocessing

Preprocessing software	*Provide detail on software version and revision number and on specific parameters (model/functions, brain extraction, segmentation, smoothing kernel size, etc.).*
Normalization	*If data were normalized/standardized, describe the approach(es): specify linear or non-linear and define image types used for transformation OR indicate that data were not normalized and explain rationale for lack of normalization.*
Normalization template	*Describe the template used for normalization/transformation, specifying subject space or group standardized space (e.g. original Talairach, MNI305, ICBM152) OR indicate that the data were not normalized.*
Noise and artifact removal	*Describe your procedure(s) for artifact and structured noise removal, specifying motion parameters, tissue signals and physiological signals (heart rate, respiration).*
Volume censoring	*Define your software and/or method and criteria for volume censoring, and state the extent of such censoring.*

Statistical modeling & inference

Model type and settings — *Specify type (mass univariate, multivariate, RSA, predictive, etc.) and describe essential details of the model at the first and second levels (e.g. fixed, random or mixed effects; drift or auto-correlation).*

Effect(s) tested — *Define precise effect in terms of the task or stimulus conditions instead of psychological concepts and indicate whether ANOVA or factorial designs were used.*

Specify type of analysis: ☐ Whole brain ☐ ROI-based ☐ Both

Statistic type for inference — *Specify voxel-wise or cluster-wise and report all relevant parameters for cluster-wise methods.*

(See Eklund et al. 2016)

Correction — *Describe the type of correction and how it is obtained for multiple comparisons (e.g. FWE, FDR, permutation or Monte Carlo).*

Models & analysis

n/a	Involved in the study
☐	☐ Functional and/or effective connectivity
☐	☐ Graph analysis
☐	☐ Multivariate modeling or predictive analysis

Functional and/or effective connectivity — *Report the measures of dependence used and the model details (e.g. Pearson correlation, partial correlation, mutual information).*

Graph analysis — *Report the dependent variable and connectivity measure, specifying weighted graph or binarized graph, subject- or group-level, and the global and/or node summaries used (e.g. clustering coefficient, efficiency, etc.).*

Multivariate modeling and predictive analysis — *Specify independent variables, features extraction and dimension reduction, model, training and evaluation metrics.*
